# Quantitative evaluation of China’s artificial intelligence policies: A PMC index-based modeling approach

**DOI:** 10.1371/journal.pone.0335423

**Published:** 2026-02-26

**Authors:** Xia Liu, Xuan Zhuang, Hongfeng Zhang, Han Zhang, Yuli Wang, Juntao Chen

**Affiliations:** 1 Faculty of Humanities and Social Sciences, Macao Polytechnic University, Macao, China; 2 Sanya Aviation and Tourism College, Sanya, China; 3 School of Business, Pusan National University, Busan, Republic of Korea; 4 Hainan College of Economics and Business, Haikou, China; Islamic World Science & Technology Monitoring and Citation Institute (ISC), IRAN, ISLAMIC REPUBLIC OF

## Abstract

With the rapid development of artificial intelligence (AI), various countries have introduced policies to address the social, economic, and ethical challenges brought by technological advancements. This study systematically evaluates the effectiveness of China’s AI policies based on the Policy Model Consistency (PMC) method and conducts a comparative analysis with policies from developed countries in Europe and the United States. By constructing a multi-dimensional quantitative assessment system that encompasses indicators such as policy types, timeliness, content, fields, evaluation, tools, and effectiveness levels, this study fills a gap in the existing research on quantitative evaluation. Text mining and high-frequency word analysis revealed the core themes and focus areas of the policies, laying the groundwork for subsequent quantitative analysis. The study finds that China’s AI policies have achieved significant results in promoting technological innovation, industrial development, and social transformation; however, shortcomings remain in legal protection, ethical regulation, cross-domain collaboration, and sustainable development issues. Further cross-national comparisons indicate that there are differences between China and developed countries in Europe and the United States in terms of AI policy design and implementation, particularly regarding the application of policy tools and the driving forces behind international collaboration. Based on the empirical analysis results using the PMC index model, this study proposes targeted policy optimization suggestions aimed at enhancing policy execution and adaptability. This study not only provides an innovative framework for the quantitative evaluation of AI policies but also offers theoretical support for the collaborative development of global AI policies.

## 1. Introduction

Artificial Intelligence (AI) technology has become an important driving force for global scientific and technological progress and economic development, profoundly affecting all walks of life, especially in the fields of industry, healthcare, education, and other areas, showing great potential for application. With the rapid development of technologies such as big data, cloud computing, and deep learning, the innovative application of AI technology is changing the operations of traditional industries and promoting the transformation of the global economic model (Brynjolfsson & McAfee, 2014; Schwab, 2016). [1,2] Governments have also considered AI technology as a strategic priority to enhance international competitiveness and have introduced policies and regulations to promote the development and application of the AI industry.

In China, artificial intelligence (AI) has been incorporated into the national strategic plan and has become an important part of the country’s innovation-driven development strategy. Since China put forward its “Artificial Intelligence 2.0” strategy in 2017, AI has played an increasingly prominent role in the country’s economic development and social governance. In 2021, the Central People’s Government of the People’s Republic of China issued the “Outline of the Fourteenth Five-Year Plan for the National Economic and Social Development of the People’s Republic of China and the Visionary Objectives for the Year 2035” further specified the goal of promoting the deep integration of AI with the real economy, and enhancing the level of intelligent manufacturing and digital services. Against this backdrop, the Chinese government has successively released several policy documents on AI technology innovation, industrial development, and social governance to promote the transformation of AI technology into practical applications and enhance national competitiveness. However, with the rapid development of AI technology, the existing policy system has gradually exposed imperfections and lagging problems in response to the challenges posed by AI technology, and there is still a gap in research on policy evaluation and implementation effects.

Although many studies have extensively discussed AI policies, most of them have focused on qualitative analysis and lack quantitative assessment of the effects of policy implementation (Yang & Huang, 2022; Niklas & Dencik, 2024) [3,4]. In China, there are still many limitations in AI policy research. First, the existing literature focuses more on the theoretical discussion of AI policies and lacks an in-depth quantitative analysis of the actual implementation effects of policies, resulting in a lack of solid data support for policy optimization. Second, although policy classification methods have been explored to some extent, research on the in-depth combination of policy tools and content and the specific problems in their implementation is still insufficient. In addition, cross-country comparative studies tend to focus on developed countries such as Europe and the United States and lack a detailed analysis of emerging economies such as China, limiting in-depth understanding of the differences and commonalities in global AI policies.

To fill the above research gaps, this study adopts the Policy Model Consistency (PMC) methodology, aiming to quantitatively assess the implementation effects of China’s AI policies, explore in depth the interaction between policy tools and policy content, and reveal the strengths and weaknesses of the policy implementation process. The PMC methodology provides a structured approach to evaluate the consistency and effectiveness of policies by constructing a policy matrix. This matrix integrates various policy tools and their corresponding implementation outcomes, allowing for a detailed and quantitative analysis of the policy implementation process. Through this method, we can systematically assess the actual effects of China’s AI policies in different areas and analyze the possible biases and shortcomings of policy implementation. In addition, this study will explore the similarities and differences between China’s AI policies and those of developed countries in Europe and the United States by combining a cross-country comparative perspective to provide a reference basis for the further optimization of China’s AI policies.

This study has several important academic and practical implications. First, by introducing the PMC method, this study provides a new systematic framework for the quantitative assessment of AI policies, filling the gap in existing research where qualitative analysis dominates. By comprehensively analyzing the policy content, implementation process and effects, this study can provide concrete data support and improvement directions for policy optimization. Second, this cross-country comparative study will further promote the understanding of the differences in AI policies in different countries and provide theoretical support for the synergistic development of global AI policies. Finally, the research in this study will help promote the responsible development of AI technology, provide a scientific basis for the formulation and optimization of national AI policies, and promote AI technology to better serve social development and economic transformation.

## 2. Literature review

### 2.1. Policy evaluation

Policy evaluation is a systematic analysis and assessment of the effectiveness and efficiency of policy implementation and its impact on society through qualitative or quantitative analysis, thus providing a basis for policy optimization. Zhu et al.(2025) [5] analyzed the overall evolution trend of AI policies in China, and although the number of policy texts increased year by year, there were still problems such as lack of innovation. Zhang et al. (2025) [6] found that the U.S. AI policy has a “government-society-market” linkage, which is characterized by internal and external “double-sided”. Wei and Li (2024) [7] quantitatively analyzed the AI policies issued by major countries around the world, and summarized the focus areas of AI policies at various stages. Zhou et al. (2025) [8] found that China’s AI data governance policies cover multiple levels, geographies, and domains after comprehensively considering the content of policy formulation and geography, etc. Yang and Huang (2022) [3] proposed a quantitative analysis of the evolution of China’s AI policies, especially the changes in policy objectives, core institutions, and they pointed out that China’s AI policies in multiple stages of the They pointed out the change of focus, including the focus on technological innovation and the initial focus on ethical issues, which is valuable for the future assessment of China’s AI policy. In addition, Niklas and Dencik (2024) [4] examined the issue of data justice and discrimination in the EU AI policy, revealing how to deal with the negative impacts of technology on society during the policy implementation process, which provides an important reference for the evaluation of the implementation effects of the policy. Evangelista (2025) [9], in analyzing the impact of generating AI tools (e.g., ChatGPT) on academic integrity, proposes a new assessment framework that aims to reduce the impact of AI-generated content on exams and assessments. This is particularly important for improving the assessment of AI policies in China’s education sector.Peng et al (2024). [10] summarized the hotspots and topic evolution paths of AI education in China, and found that “education”, “construction”, and “development” were the most important topics in the last decade. development” have been the hot topics of policies in this field over the past ten years.

In the context of evaluating the effectiveness and efficiency of policy implementation, Nordström (2022) [11] explored the uncertainty faced by the implementation of decision-making processes based on machine learning and AI techniques in public policy decision-making, noting that AI public policy decisions are highly uncertain due to the vague definition of AI, the uncertainty of implementation outcomes, and pacing issues. Public policy makers are advised to adopt argumentation methods in decision theory to mitigate this uncertainty, especially considering framing and timing strategies. Schmitt and Koutroumpis (2025) [12] investigated strategies to address cybersecurity threats using AI and targeted policy measures, emphasizing the synergy between technology and regulation. By combining federated learning, blockchain, AI-driven policies, and a zero-trust framework, a multilayered defense model is proposed to counter complex cyberattacks in cloud networks. Evangelista (2025) [9] analyzes the impact of AI technologies, such as ChatGPT, on academic integrity in higher education, and proposes to address the challenge by redesigning exams, developing assessment strategies, using AI detection tools and developing ethical policies to address the challenges in order to promote ethical use of AI in education. Michalak (2023) [13] emphasized the importance of academic librarians in developing AI policies, describing their unique skills and expertise, as well as the challenges and solutions they face. By actively involving librarians, institutions can develop comprehensive and ethical AI policies that prioritize social responsibility and respect for human rights.

To assess the impact of policies on society, Anthopoulos and Kazantzi (2022) [14] analyzed a model for urban energy efficiency assessment from the perspective of AI and big data and explored the impact of emerging technologies on urban energy systems. The study pointed out that AI and big data have important applications in urban energy efficiency assessment, but also bring new challenges such as data privacy and security issues. Danish and Senjyu (2023) [15] proposed an AI-driven circular economy policy framework for sustainable energy development. This study emphasizes the integration of multidisciplinary approaches and policy tools to cope with rapid changes in energy policy and technological advances.

### 2.2. Policy classification

Policy classification uses the content and tools of a policy to determine the type of policy. Zhou et al. (2023) [16] obtained the ethical principles of AI policies after analysis, including the principle of benefit, the principle of responsibility, and other eight principles. The study presented by Mathiyazhagan and La Fors (2023) [17] emphasized children’s right to participate in AI policies, and proposed a transnational co-creative policy approach, which provides a new perspective for ethical policies on AI. Especially when it comes to social ethics, it is important to take into account the interests of different groups. Zang et al. (2021) [18] found that there is a shortage of demand-oriented tools in China in terms of AI policy tools, while the composition of environment-oriented tools needs to be optimized and adjusted appropriately. Yang et al. (2025) [19] classify the AI regulatory policy in the United States into four core policy tools, such as the list-record style, and make suggestions for AI regulation in China. Liebig et al. (2024) [20] study the multilevel governance framework of Germany’s local AI policy, emphasizing the combination of knowledge transfer and industry cooperation, which provides a useful borrowing.

In terms of categorizing policy content, Hine and Floridi (2024) [21] provided a comparative analysis of AI policies in the US and China, exploring the differences between the two countries in terms of technological philosophies, policy goals and measures, and the reasons behind them. It has been pointed out that there are significant differences between the AI policies of the United States and China, which stem from their different technological philosophies and policy-making contexts of the two countries. Niklas and Dencik (2024) [4] focused on data justice issues in the EU’s AI policy, especially policy formulation and measures of discrimination. It found that the EU views discrimination as an inherent risk of AI and manages and evaluates this risk through procedural safeguards to support the development of a credible AI market. Schiff (2023) [22], by analyzing the national AI policy strategy of the United States, found that the instrumental value of education in supporting the AI workforce and developing more AI experts was prioritized, while the use of AI in education and its ethical implications were under-emphasized.

In terms of categorizing policy tools, David et al. (2024) [23] examined the role of local governments in AI policy-making using 16 German states as a case study. Local governments were found to play an active role in knowledge transfer, commercialization, different economic identities, and ethical principles. Khan et al. (2024) [24] provided insights into the promotion of AI policies by the US federal government through multiple policy process frameworks, emphasizing integrated strategies to address ethical, legal, and infrastructural challenges. Rönnblom et al. [25] explored gender equality in Swedish AI policy, analyzing how gender equality is expressed in policy documents. The study found that gender equality is mainly represented in Swedish AI policy as an issue of lack of knowledge and information and fails to adequately account for gender power relations.

### 2.3. Comparative study of policie

Comprehensive comparisons of policies between China and other countries were conducted using text analysis and other methods to analyze the policy focuses of different countries. Luo et al. (2025) [26] compared the AI education application policies of China, Japan, the United Kingdom, and the United States and found that the four countries have reached a consensus on talent cultivation mechanisms. Hine and Floridi (2024) [21] conducted a comparative analysis of the AI policies of China and the United States, revealing how the differences in philosophical and political systems between the two countries have shaped their respective AI policy strategies. This provides theoretical support for further comparisons of the ethical, technological, and social governance focuses on AI policies in different countries. Li et al. (2025) [27] summarize the regulatory policies and practices of China, the US, the EU and the UK in the field of generative AI, and then put forward a suggested path for China to deal with the regulation of generative AI. Pham and Davies (2024) [28] delve deeper into the issue of technological solutionism versus fundamental rights in the EU’s AI bill, analyzing how the EU how to balance marketization and ethical issues in the policy. This study compares the way of different countries to deal with AI policy and provides guidance for the improvement of China’s generative AI regulatory policy. Guo (2021) [29] compares the policy differences between China and Japan in AI and education, and affirms the important role of AI technology in education. Evangelista (2025) [9] also provides an in-depth analysis in this area, especially for how to reduce the academic integrity issues brought by AI through policy design. Wu et al. (2019) [30], on the other hand, made a comprehensive comparison of the literature studying science and technology policies in China and the United States, and found that both China and the United States have shown a high degree of attention to talent cultivation and innovation. Zhu et al. (2024) [31] used the PMC policy index to evaluate and compare the AI policies of China and the United States, and proposed an optimization path for China’s AI policies. Mao and Mei. (2020) [32] used the AI policy texts of China, the United States, Japan, the United Kingdom, and France as the object of their study, and the results showed that the policy tools of the five countries were mainly environmental and supply-oriented. Ulnicane et al. (2021) [33], in their study of the governance framework of AI policy, proposed that the government and the society should take more responsibility in the process of AI development and that through multi-party collaboration to ensure ethical and social justice in AI, which provides a new perspective to compare AI governance models in different countries.

In terms of cross-country policy comparisons, Kim et al. (2023) [34] examined South Korea’s trustworthy AI policies and compared them with those of other countries, and proposed trust-based policy recommendations to maximize the positive impacts and minimize the negative impacts of AI technologies. Robinson et al. (2020) [35] examined trust in AI policies in the Nordic countries, cultural values such as transparency and openness, and explored how these values influence the development of AI technologies in society.

In terms of the comparison of policy focus, Yang and Huang (2022) [3] analyzed the developmental evolution of China’s AI policy through bibliometric methods and identified the core policies and goals at different stages. The study found that China’s AI policy underwent a shift from technology-driven to strategic planning, emphasizing the importance of AI in national development. Diallo et al. (2025) [36], through a case study of four countries in Africa, pointed out that the global AI readiness assessment fails to adequately capture the progress of African countries, and proposed a suitable methodology for assessing AI policies in Africa. Papyshev (2024) [37] explores the use of generative AI in policy education, emphasizing the importance of developing academic policies on AI use, conducting AI training, and developing domain-specific AI systems.

### 2.4. Research summary

The existing literature provides multidimensional perspectives and in-depth analysis in the field of AI policy, covering a wide range of aspects from policy evaluation and categorization to comparative studies. Scholars have assessed the implementation effects and social impacts of AI policies through qualitative and quantitative analyses, and explored in-depth issues such as the ethics, efficiency, and transparency of policies. In terms of policy categorization, content- and tool-based categorization methods help us to understand the focus and characteristics of different policies. Cross-country comparative studies, on the other hand, reveal the similarities and differences in global AI policies, providing important references and lessons for policymakers. Although existing studies have made important progress in this area, there are still some areas that need to be further explored, especially in the comparative study of quantitative assessment and policies in emerging economies.

Although the existing research has achieved many results in the field of AI policy, there are still some key limitations. First, existing policy evaluation studies mainly focus on qualitative analysis and lack a quantitative assessment of policy implementation effects, resulting in a lack of solid data support for policy optimization. Quantitative assessments can provide more concrete evidence to help policymakers identify problems in actual implementation and adjust strategies in a timely manner. Quantitative monitoring of policy implementation can effectively improve the transparency of policy feedback and promote the continuous optimization of policies. Second, although a variety of frameworks have been proposed for policy classification, an analysis of the in-depth integration between policy tools and policy content is still insufficient. Existing research mostly focuses on defining policy objectives, ignoring the specific operational mechanisms of policy tools in actual operations. Therefore, an in-depth exploration of how policy instruments synergize with policy content is crucial for a comprehensive understanding of the implementation effects of AI policies. Most cross-country comparative studies have focused on Europe and the United States, and relatively few studies have been conducted on AI policies in emerging economies, such as China, limiting the overall vision of global AI policies. Emerging economies have their own unique challenges and needs in AI policy formulation, especially in terms of the cross-sectoral coordination of policies, driving modes of technological innovation, and concerns for social equity, which deserve more attention.

In view of these shortcomings, it is particularly important to conduct an in-depth research using the Policy Model Consistency (PMC) method, which, through the construction of policy matrices, can provide a systematic framework for policy analysis and comprehensively assess the content, implementation process, and effects of policies. It can not only clearly show the similarities and differences between different policies, but also provide specific directions and suggestions for policy optimization.The strength of the PMC method lies in its quantitative analysis capability, which can fill the gap between quantitative assessment and in-depth analysis in existing studies. In addition, by integrating perspectives from multiple disciplines, such as sociology, economics, and law, the PMC methodology can adapt to the dynamic changes in policies and ensure that policy research has both theoretical depth and meets the needs of practical applications. Especially in AI policy research in emerging economies, the PMC methodology can provide policymakers with valuable quantitative bases and specific recommendations and provide more comprehensive and in-depth analysis for AI policy research in China and other developing countries.

In summary, although existing research provides rich insights into the theory and practice of AI policy, there is still room for strengthening quantitative assessment, in-depth analysis of the combination of policy tools and content, and comparative policy research in emerging economies. Adopting the PMC methodology can not only fill the existing research gaps but also promote the development of AI policy research to a higher level, especially in providing more operational guidance in the actual policy implementation and optimization process.

## 3. Methods

### 3.1. Sources of policy texts

To ensure a comprehensive understanding of China’s AI policies, this study systematically sorts and analyzes the relevant policy documents and adopts a multi-channel search method based on the hierarchical and geographic nature of the policies to ensure that the relevant policies at the national level as well as, provincial, and municipal levels are covered. To this end, this paper follows the following steps: (1) visit the Chinese government website and other official platforms (https://www.gov.cn/, accessed on April 2025(to search for relevant policy documents and regulations on AI at the national level; (2) Log in to the “www.pkulaw.com” platform (accessed on April 2025), which is a comprehensive legal database maintained and funded by Peking University. The platform covers a wide range of legal documents, including national laws, regulations, and policies, as well as provincial and municipal-level policies. For this study, the search was conducted within the policy document collection. Use “artificial intelligence” as the search keyword, screen and download the AI policy documents at the provincial and municipal levels, and at the same time, supplement and improve the relevant policies at the national level to ensure the comprehensiveness and systematic coverage of the policies. (3) Check for omissions and make up for deficiencies in accordance with higher-level policies or legal frameworks referenced in the downloaded policy documents.

In terms of literature screening, this study established clear inclusion and exclusion criteria:

The inclusion criteria are as follows: official policy documents issued by national, provincial, or municipal governments and their departments; regulations, plans, guidance opinions, or implementation schemes with the keyword “artificial intelligence” in the title or main text; documents issued between 2015 and April 2025 that are currently valid and effective.

The exclusion criteria are as follows: outdated policies that have been declared invalid or revised; informal documents (such as meeting minutes, approval letters, internal notices, etc.); documents that do not involve the core content of artificial intelligence and only mention it incidentally; duplicate versions of policy texts.

To ensure the quality of the screening process, this study adopted a rigorous screening procedure, fully overseen by a senior public policy researcher to guarantee the scientific validity and accuracy of the results.

Preliminary Screening: The senior researcher first conducted a preliminary screening of the collected policy documents. At this stage, judgments were primarily based on the titles and abstracts of the documents. Through professional discernment and identification, documents relevant to the research topic were selected. During this process, deduplication tools were used to remove duplicates, ensuring the independence and validity of the screening results.

Full-Text Screening: Building on the preliminary screening, the senior researcher conducted a full reading and analysis of the selected documents. Drawing on extensive policy analysis experience, the researcher meticulously examined the complete content of each document, assessing its relevance to the research topic and its contribution to the research questions. The aim of this stage was to thoroughly tap into the value of the documents and to ensure that the selected documents could provide a solid foundation for subsequent research.

Final Validation: After completing the full-text screening, the senior researcher compared and validated the results against higher-level policy frameworks. Through this process, any potential omissions were identified and addressed, ensuring that the selected policy documents had integrity and consistency in terms of policy hierarchy, and could comprehensively cover the relevant policy content required for the research.

The retrieved policies were screened, policy texts that were no longer valid or of weak relevance to the topic were excluded, informal documents such as letters and approvals were removed, and 115 AI policy texts were finally selected, totaling more than 840,000 words. The selected policy texts cover national-, provincial-, and municipal-level policies, and the types of policies include notices, opinions, approaches, and programs and so on.

### 3.2. Policy text analysis

The analysis focused on the main body of 115 policy documents, excluding headers, footers, and annexes. Specific emphasis was placed on sections such as policy objectives, implementation measures, and strategic guidelines, as these parts are most likely to contain key information related to artificial intelligence policies. All documents were in ANSI-encoded TXT format and were directly imported into ROSTCM6 software (Version 6.0) for analysis without the need for additional conversion.

The TXT files were imported into ROSTCM6 software using its batch import function. Each document was assigned a unique identifier to facilitate tracking and analysis. The preprocessing steps included the following: tokenization using the software’s built-in Chinese word segmentation algorithm to split the text into individual words; text normalization, which involved converting all characters to their simplified form and removing extraneous spaces or special characters; application of a customized stop-word list to exclude common but meaningless words such as “development”, “strengthen”, and “advance”; and part-of-speech tagging to exclude conjunctions, prepositions, and other non-content words, thereby ensuring the accuracy of the analysis results.

The word frequency counting function of ROSTCM6 software was utilized to calculate the frequency of each word in the corpus, thereby identifying the most frequently occurring terms. The algorithm settings included the following: a minimum frequency threshold of 5 to filter out words that appear very infrequently; and a word length filter to exclude words with fewer than two characters, ensuring the practicality of the analysis results.

Key topics were extracted based on word frequency and contextual information to reveal the main issues and focal points in the policy documents. The number of topics extracted was set to 10, with topic weights calculated according to word frequency and contextual relevance. Concurrently, a semantic network was constructed by analyzing the co-occurrence frequency and contextual relationships of words. A co-occurrence threshold of 10 was set, and a semantic network diagram was generated to intuitively display the connection strength and network structure between words.

#### 3.2.1. Thematic analysis.

The 115 policy documents were merged and imported into *ROSTCM6* software for word separation and word frequency counting, and meaningless words such as “generation,” “strengthen” and “advance” were excluded to obtain the following high-frequency words of AI policies.

#### 3.2.2. Semantic web analysis.

Semantic network analysis graphically displays the interrelationships between high-frequency words in a policy, with the aim of explaining the structural and semantic links between different concepts in a text. In this context, high-frequency words in a text are represented by nodes and the relationships between high-frequency words are represented by edges. By analyzing these connecting relationships, the core themes and key messages of the text can be revealed. In particular, the degree centrality between nodes can be used as a criterion to judge the importance of the node in the whole network; if the number of edges connected to a node is higher, the higher the degree centrality of the node, and the more the high-frequency word is a core word in the policy.

### 3.3. PMC index modeling

The PMC index model is an analytical approach for policy modeling and evaluation that uses different theories, quantitative or qualitative models, and technical tools to measure the coherence of the policy process and the inherent strengths and weaknesses of the policies. This theoretical foundation is derived from the Omnia-Mobilis hypothesis, which reveals the intrinsic relationships and effects of policies through multidimensional analysis (Ruiz Estrada et al., 2008) [38]. In recent years, the PMC index has been applied to the evaluation of various policies and has become an important tool in the field of policy analysis and evaluation [(Xia, 2024; Zhang et al.,2025; Cui and Wang, 2024; Cai et al.,, 2024) [39–42].

The model construction process is divided into five steps: (1) sample policy selection, (2) variable selection and parameterization, (3) construction of a multi-input-output table, (4) PMC index calculation, and (5) PMC surface plotting.

#### 3.3.1. Selection of sample policies.

The PMC model requires comprehensive consideration of all relevant variables and avoids subjective biases influencing the results. Therefore, this study excludes objective factors such as the effectiveness level, issuing authority, and policy type when selecting policy samples to maximize the reliability of the results. In this study, considering the time of issuance and number of policy citations, 14 documents were selected as sample policies from 115 AI policies, as shown in Table 1.

**Table 1:** Sample policies.

Policy Number	Policy name	Publication date	Subject of the document
P1	New Generation Artificial Intelligence Ethics Standards	2021.9.25	National New Generation Artificial Intelligence Governance Professional Committee
P2	Guidelines for the Construction of the National Comprehensive Standardization System for Artificial Intelligence Industry (2024 Edition)	2024.6.5	Ministry of Industry and Information Technology, Office of the Central Cybersecurity and Informatization Commission, National Development and Reform Commission, General Administration of Quality Supervision, Inspection and Quarantine
P3	Guidelines for the Construction of the National Standard System for New Generation Artificial Intelligence	2020.7.27	General Administration of Quality Supervision, Inspection and Quarantine, Office of the Central Cyberspace Affairs Commission, National Development and Reform Commission, Ministry of Science and Technology, Ministry of Industry and Information Technology
P4	Development Plan for the New Generation of Artificial Intelligence	2017.7.8	State Council
P5	Opinions on Promoting Discipline Integration and Accelerating Graduate Education in the Field of Artificial Intelligence at “Double First-Class” Universities	2020.1.21	Ministry of Education, National Development and Reform Commission
P6	Artificial Intelligence Innovation Action Plan for Higher Education Institutions	2018.4.2	Ministry of Finance
P7	Guidelines for Graduate Training in the Field of Artificial Intelligence (Trial)	2022.7.27	Ministry of Education
P8	Guiding Opinions on Accelerating Scenario Innovation and Promoting High-level Applications of Artificial Intelligence to Drive High-quality Economic Development	2022.7.29	Ministry of Education
P9	Guidelines for the Construction of the National New Generation Artificial Intelligence Innovation Development Pilot Zone (Revised Edition)	2020.9.29	Ministry of Science and Technology, Ministry of Education, Ministry of Industry and Information Technology
P10	Guidelines for the Construction of National New Generation Artificial Intelligence Open Innovation Platforms	2019.8.1	Ministry of Transport, Ministry of Agriculture and Rural Affairs, National Health Commission
P11	Notice on Supporting the Construction of Demonstration Application Scenarios for the New Generation of Artificial Intelligence	2022.8.12	Ministry of Science and Technology
P12	Cybersecurity Standard Practice Guide — Ethical and Security Risk Prevention for Artificial Intelligence	2021.1.5	Ministry of Science and Technology
P13	Interim Measures for the Management of Generative Artificial Intelligence Services	2023.7.10	Ministry of Science and Technology
P14	Opinions on Regulating and Strengthening the Judicial Application of Artificial Intelligence	2022.12.8	Secretariat of the National Information Security Standardization Technical Committee

#### 3.3.2. Selection of variables and parameterization.

**Fig 1:**
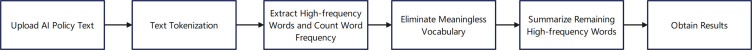
Variable selection process. The process encompasses six academically rigorous steps.

Based on the research of Ruiz Estrada et al. (2008) [38], this study selected nine Level 1 indicators, which comprehensively covered the process of policy design, implementation, and evaluation from multiple dimensions. In this paper, ROSTCM6 is used as the text analysis software to count the high-frequency words in the text after the text content is processed by lexical segmentation, and the meaningless words such as “improve” are eliminated to obtain the secondary indicators of “policy content” and “policy focus.” The secondary indicators of “policy content” and “policy focus were obtained. The operational process is illustrated in Fig 1. Seven other primary indicators and their secondary indicators were constructed based on existing studies, as shown in Table 2.

**Table 2:** Selection of indicators and parameter settings.

Primary Indicator	Secondary Indicator		Parameter Settings	Indicator Source
$X_{\text{1}}$ Policy Type	$X_{1:1}$	Prediction	Whether the policy predicts development prospects.	Ruiz Estrada et al. (2008) [38]
	$X_{1:2}$	Suggestion	Whether the policy makes development proposals.	
	$X_{1:3}$	Regulation	Whether the policy contains regulatory measures.	
	$X_{1:4}$	Support	Whether or not the policy provides support initiatives for development.	
	$X_{1:5}$	Guidance	Whether the policy serves to guide development.	
	$X_{1:6}$	Diagnosis	Whether the policy summarizes the current state of development.	
$X_{\text{2}}$ Policy Timeliness	$X_{2:1}$	Short-term	Whether the policy is short-term (less than 3 years).	Ruiz Estrada et al. (2008) [38]
	$X_{2:2}$	Medium-term	Whether the time limit of the policy is short-term (3–5 years).	
	$X_{2:3}$	Long-term	Whether the policy’s statute of limitations is short-term (more than 5 years).	
$X_{\text{3}}$ Policy Content	$X_{3:1}$	Scientific Innovation	Whether the policy element includes scientific innovation.	Formulate based on high-frequency words
	$X_{3:2}$	Talent Development	Whether the policy content includes talent development.	
	$X_{3:3}$	Ecological Construction	Whether the content of this policy includes the construction of industrial ecology, yes is 1, no is 0	
	$X_{3:4}$	Cooperation and Communication	Whether the policy includes cooperative exchanges.	
	$X_{3:5}$	Public Service	Whether the policy element includes public services.	
	$X_{3:6}$	Data Security	Whether the policy content includes data security.	
$X_{\text{4}}$ Policy Field	$X_{4:1}$	Politics	Whether the policy is in the political sphere.	Zhu et al. (2024) [31]
	$X_{4:2}$	Economy	Whether the policy relates to the economic sphere.	
	$X_{4:3}$	Society	Whether the policy addresses the social sphere.	
	$X_{4:4}$	Environment	Whether the policy addresses the environmental area.	
	$X_{4:5}$	Science and Technology	Whether the policy relates to the field of science and technology.	
	$X_{4:6}$	Culture	Whether the policy relates to the field of culture.	
$X_{\text{5}}$ Policy Evaluation	$X_{5:1}$	Sufficient Basis	Whether the development of the policy is well-founded.	Song et al. (2021) [43]
	$X_{5:2}$	Clear Objectives	Whether the policy contains clear development objectives.	
	$X_{5:3}$	Detailed Planning	Whether the policy is well planned.	
	$X_{5:4}$	Scientific Proposal	Whether the program of the policy is scientifically sound.	
$X_{\text{6}}$ Policy Object	$X_{6:1}$	Government	Whether the target of the policy role involves the government.	Song et al. (2021) [43]
	$X_{6:2}$	Enterprise	Whether the policy is targeted at enterprises.	
	$X_{6:3}$	Public	Whether the policy is aimed at the public.	
	$X_{6:4}$	Non-profit Organizations	Whether the policy’s targeting involves non-profit organizations.	
$X_{\text{7}}$ Policy Tool	$X_{7:1}$	Supply-driven	Whether the policy is supply-based.	Mao et al. (2020) [32]
	$X_{7:2}$	Demand-driven	Whether the policy is demand-based.	
	$X_{7:3}$	Environment-driven	Whether the policy is environmental.	
$X_{\text{8}}$ Policy Focus	$X_{8:1}$	Technology Application	Whether the focus of the policy includes the application of technology.	Ruiz Estrada et al. (2008) [38] Formulate based on high-frequency words
	$X_{8:2}$	Industry Development	Whether the focus of the policy includes industrial development.	
	$X_{8:3}$	Resource Construction	Whether the focus of the policy includes resource building.	
	$X_{8:4}$	Intelligent System	Whether the focus of the policy includes smart system building.	
$X_{\text{9}}$ Policy Effectiveness	$X_{9:1}$	Laws and Regulations	Whether the policy is a law or regulation, yes is 1, no is 0	Zhu et al. (2024) [31]
	$X_{9:2}$	Administrative Regulations	Whether the policy is an administrative regulation.	
	$X_{9:3}$	Departmental Rules	Whether the policy is a departmental regulation.	
	$X_{9:4}$	Normative Documents	Whether the policy is a normative document.	
	$X_{9:5}$	Industry Regulations	Whether the policy is an industry requirement.	

By introducing variables such as “policy type,” “policy duration,” “policy area,” etc., it is possible to analyze in depth whether the policy has an impact on the society and the economy and whether the impact is long-term, in terms of the means of intervention, the period of implementation, the area of action, and so on. Through textual analysis of the policy, the “policy content” is determined from the perspective of the sustainable development of the AI industry. At the same time, the variables of “policy tool,” “policy object” and “policy focus” are set to reveal the specific path of policy implementation, audience groups and their strategic directions of concern, so as to To assess the specific implementation path of the policy and the driving force of the focus areas. To ensure the scientific nature and implementation strength of the policy, the model also includes the variables of “policy evaluation” and “effectiveness level to judge the clarity of the policy objectives, the details of the plan, and its legal effect. In summary, the combined use of the above variables enables the PMC policy index model to comprehensively and systematically assess the multidimensional impact of policies, providing a more scientific and precise basis for policy optimization and decision-making.

The Secondary Indicators under each Primary Indicator are of equal importance; therefore, when assigning values to the Secondary Indicators, binary values of 0 and 1 are used. If the AI policy contains the content of the Secondary Indicator, then the parameter of the Secondary Indicator is denoted as 1, otherwise, it is denoted as 0,as shown in Table 3.

**Table 3:** Multi-input-output table.

Primary Indicator	Secondary Indicator
$X_{\text{1}}$	$X_{1:1}$ $X_{1:2}$ $X_{1:3}$ $X_{1:4}$ $X_{1:5}$ $X_{1:6}$
$X_{\text{2}}$	$X_{2:1}$ $X_{2:2}$ $X_{2:3}$
$X_{\text{3}}$	$X_{3:1}$ $X_{3:2}$ $X_{3:3}$ $X_{3:4}$ $X_{3:5}$ $X_{3:6}$
$X_{\text{4}}$	$X_{4:1}$ $X_{4:2}$ $X_{4:3}$ $X_{4:4}$ $X_{4:5}$ $X_{4:6}$
$X_{\text{5}}$	$X_{5:1}$ $X_{5:2}$ $X_{5:3}$ $X_{5:4}$
$X_{\text{6}}$	$X_{6:1}$ $X_{6:2}$ $X_{6:3}$ $X_{6:4}$
$X_{\text{7}}$	$X_{7:1}$ $X_{7:2}$ $X_{7:3}$
$X_{\text{8}}$	$X_{8:1}$ $X_{8:2}$ $X_{8:3}$ $X_{8:4}$
$X_{\text{9}}$	$X_{9:1}$ $X_{9:2}$ $X_{9:3}$ $X_{9:4}$ $X_{9:5}$

#### 3.3.3. Construction of multi-input-output tables.

A multi-input–output table is a database analysis framework that uses binary data to assign values to secondary indicators. If the policy contained the content of the secondary indicator, it was recorded as 1; otherwise, it was recorded as 0. Assigning a value to each secondary indicator produces the multi-input-output table shown in Table 3, which is expressed as a 0–1 matrix.

#### 3.3.4. Calculation of PMC index.

The calculation of the PMC index is divided into four steps: (1) Assign the 9 first-level indicators and 41 second-level indicators to the multi-input-output table; (2) Referring to Equations 1 and 2, analyze the AI policy using the text mining method, and assign the value of each second-level indicator according to the first-level indicators one by one; (3) Calculate the value of each first-level indicator using Equation 3. That is, the results of assigning values to all second-level indicators under first-level indicators are summed and then divided by the number of second-level indicators; and (4) The PMC index is calculated using Equation 4. and the PMC index is equal to the sum of the values of all first-level indicators.


X~N[0,1] (1)

X={XR:[0,1]} (2)

$$X_{t}\left[\sum_{j=1}^{n}\frac{X_{tj}}{T\left(X_{tj}\right)}\right]\text{\textbackslash{}hspace\{1em}\}t=1,2,3,...$$ (3)

$$PMC=\left[\begin{array}{c} @l@X_{\text{1}}\left(\sum_{i=1}^{\text{6}}X_{1i}/6\right)+X_{\text{2}}\left(\sum_{j=1}^{\text{3}}X_{2j}/3\right)+X_{\text{3}}\left(\sum_{k=1}^{\text{6}}X_{3k}/6\right)+\\ X_{\text{4}}\left(\sum_{l=1}^{\text{6}}X_{4l}/6\right)+X_{\text{5}}\left(\sum_{m=1}^{\text{4}}X_{5m}/4\right)+X_{\text{6}}\left(\sum_{n=1}^{\text{4}}X_{6n}/4\right)+\\ X_{\text{7}}\left(\sum_{o=1}^{\text{3}}X_{7o}/3\right)+X_{\text{8}}\left(\sum_{p=1}^{\text{4}}X_{8p}/4\right)+X_{\text{9}}\left(\sum_{r=1}^{\text{5}}X_{9r}/5\right) \end{array}\right]$$ (4)

Finally, the analysis of the PMC index results depend on the consistency of the study at the four levels, as shown in Table 4. If the PMC index of the policy is between eight and nine, the policy has perfect consistency. If the PMC index of the policy is between six and eight, the policy has good consistency. If the PMC index of the policy is between four and six, the policy has acceptable consistency. If the PMC index of the policy is less than four, the consistency is low.

**Table 4:** Policy hierarchy.

PMC Index	PMC<4	4≤PMC<6	6≤PMC<8	8≤PMC≤9
Policy Level	Unqualified	Qualified	Good	Excellent

#### 3.3.5. PMC surface construction.

After calculating the PMC index, the results of each policy’s first-order index calculation are written into a 3rd-order matrix as shown in Equation 5, and the PMC surface is constructed using this matrix, which can graphically visualize the degree of merit of the policy’s performance.


$$PMC=\left[\begin{array}{ccc} @ccc@X_{\text{1}} & X_{\text{2}} & X_{\text{3}}\\ X_{\text{4}} & X_{\text{5}} & X_{\text{6}}\\ X_{\text{7}} & X_{\text{8}} & X_{\text{9}} \end{array}\right]$$ (5)

## 4. Results

The high-frequency words of the AI policies are listed in Table 5. From the data in Table 1, it can be found that the focus of the current AI policy is mainly on the following aspects: First, technological innovation and application is the key driving force of the policy. With the rapid development of science and technology, the policy focuses on promoting technological research and development and the landing of innovative applications, particularly in the promotion of specific application scenarios such as intelligentization and “wisdom +”. Second, the policy encourages cross-industry and cross-border exchanges and cooperation in AI and promotes synergistic development of the AI industry in multiple fields. In addition, the policy also focuses on standardization construction and security, promotes the standardization of AI technology, and strengthens the supervision of data security issues. Finally, the policy encourages the establishment of a sound talent training system and education mechanism as well as the promotion of the benign development of AI industrial ecology through demonstration projects and capacity building.

**Table 5:** Artificial intelligence policy high frequency words.

High-frequency words	Word frequency	High-frequency words	Word frequency	High-frequency words	Word frequency
Artificial Intelligence (AI)	8136	System	1103	Standard	672
Intelligence/ Smart	5553	Integration	1096	Development	669
Technology	4301	Science and Technology	1095	Healthcare/ Medical	668
Application	4130	Scenario	1075	Policy	636
Enterprise/ Company	3071	Intelligence/ Intelligentization	1004	Economy	616
Innovation	3023	Project	987	Environment	612
Field/ Domain	2299	Center	978	Computing	607
Service	2297	Open	915	Ecology	599
Platform	2236	Nation/ Country	915	Universities/ Higher Education Institutions	587
Data	2181	Capability	872	Cooperation	567
Artificial	1440	Security	851	Cultivation/ Training	550
Talent/ Personnel	1317	Level	771	Facilities	549
Foundation/ Basis	1314	Demonstration	762	Finance	549
Research	1240	Cultivation	760	Agriculture	541
Research and Development (R&D)	1217	Collaboration/ Coordination	757	Robotics	540
Wisdom/ Intelligent	1216	Construction/ Building	747	Model	522
Model	1165	Institution	700	Depth	521
Resources	1149	Education	692	Informatization	511
Big Data	1141	Industry	678	Demand	508
Management	1104	Society	674	Mechanism	508

**Fig 2:**
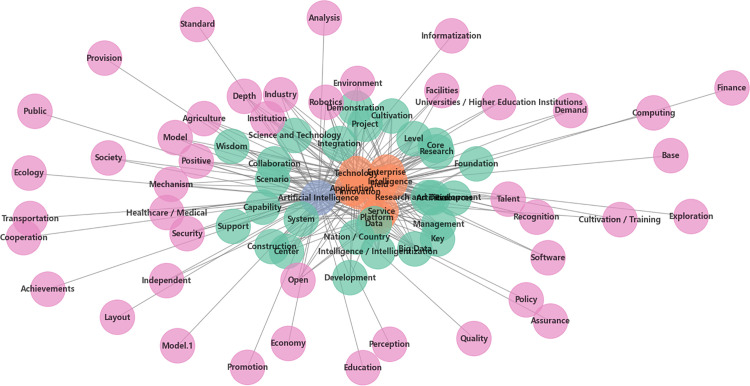
Semantic network analysis. The green cluster centers on science and technology research and development, resource integration, and intelligentization construction at the national or regional level.The orange cluster focuses on the application of intelligent technologies in the fields of enterprises, innovation, and services.The blue cluster is dedicated to the core domain of artificial intelligence.The pink cluster encompasses multiple aspects, including talent development, education, industry, and policy.

Semantic network analysis of the text to obtain Fig 2. The network graph presents a multilevel, cross-domain structure that shows a broad coverage of AI policies. From Fig 1, it can be observed that AI is the center node in the semantic network with the highest connectivity and is the core of the policy. The next ones with high connectivity are technology, application, intelligence, and innovation, indicating that they are the key factors driving the development of AI. In addition, fields, research, platforms, etc. also have high degrees, highlighting the importance of AI policies in multidisciplinary collaboration, research and innovation, and platform construction. This suggests that, in addition to technology and applications, the policy framework for AI also focuses on promoting cross-field collaboration, strengthening basic research and technology development, and supporting the promotion and application of technology through the establishment of a sound platform.

The quantitative evaluation of AI policies based on the above PMC index model consists of three main stages: PMC index calculation, PMC surface drawing, and the quantitative evaluation of policies.

### 4.1. Calculation of PMC index

The PMC index was calculated for the 14 selected AI policies and the results are presented in Table 6. Policy P4 has the best performance among all level 1 variables, with a score of 1.00, reflecting its comprehensive and systematic nature in terms of policy type, timeliness, content, and tools. Policy P11, on the other hand, had the lowest PMC index, especially scoring low on key features, reflecting its deficiencies. Overall, the policies show strong diversity and effectiveness in terms of policy tools (average score of 0.88) and policy content (0.76) but are relatively deficient in terms of attention to policy objects (0.41). This study not only quantitatively evaluates the performance of different AI policies but also provides a clear direction for their subsequent optimization and promotes a deeper understanding of the effectiveness of policy implementation.

**Table 6:** PMC indices for the sample policies.

Primary Variable	X1	X2	X3	X4	X5	X6	X7	X8	X9
P1	0.50	1.00	0.67	0.67	1.00	1.00	0.67	0.25	0.40
P2	0.67	1.00	0.83	0.67	0.75	0.75	1.00	1.00	0.40
P3	0.67	0.33	0.67	1.00	0.75	0.75	0.67	1.00	0.40
P4	1.00	1.00	1.00	1.00	0.75	1.00	1.00	1.00	0.40
P5	0.83	1.00	0.67	0.67	1.00	0.75	0.33	0.50	0.40
P6	0.83	1.00	0.83	0.50	1.00	1.00	1.00	1.00	0.40
P7	0.83	0.33	0.83	0.50	1.00	0.75	0.33	0.75	0.40
P8	0.67	1.00	1.00	0.83	1.00	0.75	1.00	1.00	0.40
P9	0.83	0.67	0.83	0.83	1.00	0.75	1.00	1.00	0.40
P10	0.67	0.33	0.67	0.67	0.75	1.00	0.67	0.75	0.40
P11	0.50	0.33	0.50	1.00	0.75	0.75	0.33	0.75	0.40
P12	0.67	1.00	0.67	0.67	0.50	0.75	0.67	0.75	0.40
P13	0.67	1.00	0.83	0.67	1.00	1.00	0.67	1.00	0.60
P14	0.83	1.00	0.67	0.50	1.00	0.25	0.67	1.00	0.40
average value	0.73	0.79	0.76	0.73	0.88	0.80	0.71	0.84	0.41

The policies were rated and ranked according to the size of the PMC index, as shown in Table 7. Policy P4 ranked first with a score of 8.15 and was rated as “excellent,” showing its significant advantage in terms of overall capacity. Policies P6 and P8 ranked the third and the second, respectively, and were both rated “good,” indicating that these policies performed well in a number of dimensions. Most policies (P1, P2, P3, P5, P9, P12, and P13) were rated as’ good, demonstrating the overall adaptability and effectiveness of the policies. Policies P7, P10, and P11, however, scored lower, ranking 13th, 12th and 14th, respectively, with a “pass” rating, reflecting deficiencies in some of the key features of these policies. Overall, the average value for all policies is 6.65, indicating that the sample of policies as a whole performs “well” but needs to be optimized and improved in some areas.

**Table 7:** Ranking and ranking of sample policies.

Policy Number	PMC Index	Ranking	Policy Level
P1	6.15	9	Good
P2	7.07	6	Good
P3	6.23	8	Good
P4	8.15	1	Excellent
P5	6.15	10	Good
P6	7.57	3	Good
P7	5.73	13	Qualified
P8	7.65	2	Good
P9	7.32	5	Good
P10	5.90	12	Qualified
P11	5.32	14	Qualified
P12	6.07	11	Good
P13	7.43	4	Good
P14	6.32	7	Good
average value	6.65		Good

### 4.2. PMC surface plotting

**Fig 3:**
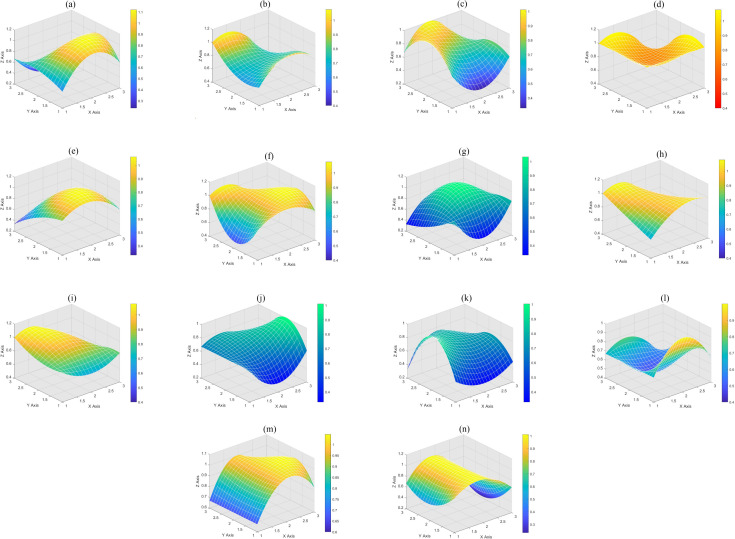
PMC surface diagram. (a) is the surface diagram of p1, (b) is the surface diagram of p2, (c) is the surface diagram of p3, (d) is the surface diagram of p4, (e) is the surface diagram of p5, (f) is the surface diagram of p6, (g) is the surface diagram of p7, (h) is the surface diagram of p8, (i) is the surface diagram of p9, (j) is the surface diagram of p10, (k) is the surface diagram of p11, (l) is the surface diagram of p12, (m) is the surface diagram of p13, and (n) is the surface diagram of p14.


The PMC index of the 14 policies are represented by a third-order matrix (Equation 6), and the surface is plotted using MATLAB to visually determine the degree of the strengths and weaknesses of the policies, as shown in Fig 3. The excellent policies use “autumn” color, the good policies use “parula” color, and the passing policies use “winter” color.

$$\begin{array}{ccc} PMC_{P1}=\left[\begin{array}{ccc} @ccc@0.50 & 1.00 & 0.67\\ 0.67 & 1.00 & 1.00\\ 0.67 & 0.25 & 0.40 \end{array}\right] & PMC_{P2}=\left[\begin{array}{ccc} @ccc@0.67 & 1.00 & 0.83\\ 0.67 & 0.75 & 0.75\\ 1.00 & 1.00 & 0.40 \end{array}\right] & PMC_{P3}=\left[\begin{array}{ccc} @ccc@0.67 & 0.33 & 0.67\\ 1.00 & 0.75 & 0.75\\ 0.67 & 1.00 & 0.40 \end{array}\right]\mathrm{\backslash vspace}1.5mm\\ PMC_{P4}=\left[\begin{array}{ccc} @ccc@1.00 & 1.00 & 1.00\\ 1.00 & 0.75 & 1.00\\ 1.00 & 1.00 & 0.40 \end{array}\right] & PMC_{P5}=\left[\begin{array}{ccc} @ccc@0.83 & 1.00 & 0.67\\ 0.67 & 1.00 & 0.75\\ 0.33 & 0.50 & 0.40 \end{array}\right] & PMC_{P6}=\left[\begin{array}{ccc} @ccc@0.83 & 1.00 & 0.83\\ 0.50 & 1.00 & 1.00\\ 1.00 & 1.00 & 0.40 \end{array}\right]\mathrm{\backslash vspace}1.5mm\\ PMC_{P7}=\left[\begin{array}{ccc} @ccc@0.83 & 0.33 & 0.83\\ 0.50 & 1.00 & 0.75\\ 0.33 & 0.75 & 0.40 \end{array}\right] & PMC_{P8}=\left[\begin{array}{ccc} @ccc@0.67 & 1.00 & 1.00\\ 0.83 & 1.00 & 0.75\\ 1.00 & 1.00 & 0.40 \end{array}\right] & PMC_{P9}=\left[\begin{array}{ccc} @ccc@0.83 & 0.67 & 0.83\\ 0.83 & 1.00 & 0.75\\ 1.00 & 1.00 & 0.40 \end{array}\right]\mathrm{\backslash vspace}1.5mm\\ PMC_{P10}=\left[\begin{array}{ccc} @ccc@0.67 & 0.33 & 0.67\\ 0.67 & 0.75 & 1.00\\ 0.67 & 0.75 & 0.40 \end{array}\right] & PMC_{P11}=\left[\begin{array}{ccc} @ccc@0.50 & 0.33 & 0.50\\ 1.00 & 0.75 & 0.75\\ 0.33 & 0.75 & 0.40 \end{array}\right] & PMC_{P12}=\left[\begin{array}{ccc} @ccc@0.67 & 1.00 & 0.67\\ 0.67 & 0.50 & 0.75\\ 0.67 & 0.75 & 0.40 \end{array}\right]\mathrm{\backslash vspace}1.5mm\\ PMC_{P13}=\left[\begin{array}{ccc} @ccc@0.67 & 1.00 & 0.83\\ 0.67 & 1.00 & 1.00\\ 0.67 & 1.00 & 0.60 \end{array}\right] & PMC_{P14}=\left[\begin{array}{ccc} @ccc@0.83 & 1.00 & 0.67\\ 0.50 & 1.00 & 0.25\\ 0.67 & 1.00 & 0.40 \end{array}\right] & \end{array}$$ (6)

### 4.3. Quantitative evaluation of policies

Among the PMC index of all policies, the index values of most policies were in the good range, with an average value of 6.65, showing that the overall policy effect was relatively positive. The policies were ranked according to the calculation of the PMC index of the 14 policies, and the ranking results were obtained, as shown in Table 7. The AI policies are categorized into three grades according to the PMC index: (1) Grade I policies. with a PMC index between 8–9, the overall performance of the policies is excellent, such as P4, and (2) Grade II policies. with a PMC index between 6–7.9, the performance of the policies is good, such as P1, P2, P3, and (3) Grade III policies. with a PMC index between 4–5.9, the performance of the policies passes, such as P7, P10, and P3. such as P7, P10, P11.

**Fig 4:**
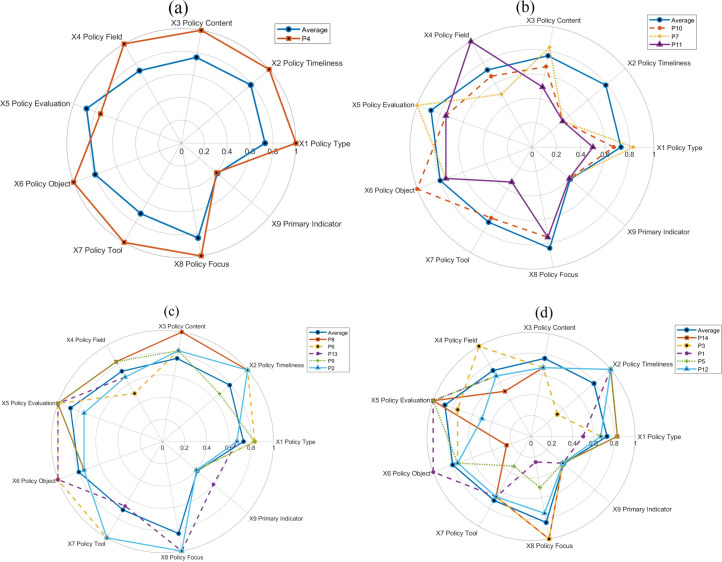
Policy radar map. (a) depicts the “Policy of excellence,” which shows outstanding performance across all evaluation dimensions, exemplified by policy P4. (b) represents “Qualified policies,” which meet basic requirements, as indicated by policies P7, P10, and P11. (c) illustrates “Good policies (part 1),” which perform well in certain key dimensions, such as policies P2, P6, P8, P9, and P13. (d) presents “Good policies (part 2),” which also demonstrate strong performance in specific dimensions, including policies P1, P3, P5, P12, and P14.

Policies are visualized in radar charts according to excellent, good, and qualified policies, respectively, as shown in Fig 4. Because there are relatively more good policies, the good policies are divided into two radar charts according to the order of scores. As shown in Fig 4, seven Primary Indicators of excellent policies were much larger than the average value, and the two Primary Indicators were slightly lower than the average value. By contrast, most of the Primary Indicator values of the qualified policies are much smaller than the average value. Most Primary Indicator values for good policies were close to the average.

Combined with Fig 4, from the perspective of the first-level indicators, the 14 policies have the highest average scores in terms of policy evaluation, which indicates that the policies have a strong policy basis and implementation guarantee and are feasible. Second, the average value of the scores for policy object and policy focus is also above 0.8, indicating that the policies have fully considered the direction of implementation and participation of multiple subjects in planning, highlighting the importance of the policies in promoting technological progress and industrial transformation. However, the overall effectiveness level of these policies is low, relying mainly on normative documents and industry regulations, of which only one policy is departmental regulation. Thus, legal protection is relatively weak, limiting the binding force and authority of policy implementation. In addition, the types of policies, policy timeliness, policy content, policy areas, and policy tools were all at a medium level. Although the policy design covers a wide range of areas and tools, there is still room for further optimization in terms of the in-depth excavation of specific areas and application of tools. Moreover, some policies have a single objective, and fail to fully consider the interactive effects of multiple dimensions and long-term sustainability when formulating policies.

## 5. Discussion

### 5.1. Overview of policy evaluation

#### 5.1.1. Graded evaluation of policies.

The policies are categorized into three levels based on their PMC index and other characteristics. Level I policies, exemplified by P4, are characterized by high authority and influence, as well as comprehensiveness and systemic nature. P4, issued by the State Council, aligns with Brynjolfsson and McAfee’s (2014) [1] view that policy should play a supportive and guiding role in the development of high technology. It guides and promotes the in-depth integration of AI in various fields, clarifies the three-step development strategy of AI, and supports scientific and technological innovation, talent cultivation, and cross-field and cross-border cooperation. However, some scholars (Zhu et al., 2025) [5] argue that more flexible contingency measures are needed to maintain the policy’s effectiveness and foresight in an uncertain environment. The relatively smooth overall trend of the PMC surface plot of P4, despite the depression due to low effectiveness, reflects the possible imbalance in its implementation (Schwab, 2016) [2].

Level II policies, including P1, P2, P3, P5, P6, P8, P9, P12, P13, and P14, show varied performances. P1 focuses on normative constraints for AI development, emphasizing data safety and ethical literacy, which is in line with the findings of Niklas and Dencik (2024) [4] on the importance of policy ethics and safety in AI. P2, jointly issued by multiple ministries, aims to build a comprehensive standardization system for the AI industry but has limitations in effectiveness and legal binding (Zhou et al., 2025) [8]. P3 highlights cross-domain integration and industrial upgrading but lacks a regulatory strategy (Schmitt and Koutroumpis, 2025) [12]. P5 encourages cooperation between governments, enterprises, and universities, while P6, issued by the Ministry of Education, focuses on talent cultivation and the construction of an intelligent system, echoing David et al.‘s (2024) [23] view on the important role of education policy in technological innovation. P8, with a strong strategic approach, promotes technological innovation and intelligent development but pays less attention to sustainable development and lacks regulatory measures for AI applications (Danish & Senjyu, 2023) [15]. P9 has clear objectives and detailed planning, supporting the construction of a good ecology for AI and enhancing AI innovation capabilities (Zhou et al., 2022) [16]. P12, like P1, focuses on preventive measures for ethical security risks but needs improvement in other aspects (Khan et al., 2024) [24]. P13, jointly issued by multiple departments, aims at the innovative development and application of AI while considering data security but lacks specific requirements in some other aspects.

Level III policies, such as P7, P10, and P11, are qualified. P7 focuses on cultivating postgraduate students in AI and provides specific regulatory measures, but its policy content is more targeted at talent cultivation rather than the ecological construction of AI (Luo et al., 2025) [26]. P10 emphasizes technological applications and industrial development but omits public service and data security components (Mathiyazhagan & La Fors, 2023) [17]. P11 aims to promote high-level application of AI technologies in multiple industry sectors but lacks development proposals and regulatory measures, and its implementation may be more complex (Ulnicane et al., 2021) [33].

#### 5.1.2. Relationship between PMC index and institutional missions.

The PMC index status appears to be influenced by the mission of the institution issuing the policy. For instance, policies issued by the State Council (e.g., P4) tend to have higher PMC indices, reflecting their comprehensive and strategic nature. These policies are designed to guide and promote the overall development of AI technology, aligning with the State Council’s mission to set national strategies and policies that have a broad and long-term impact on the country’s technological and economic development (Yang & Huang, 2022) [3]. In contrast, policies issued by specific ministries or departments (e.g., P2 by the Ministry of Industry and Information Technology) may focus more narrowly on industry-specific goals, such as building a standardization system for the AI industry. While these policies are important for their respective domains, they may lack the same level of comprehensiveness and long-term vision as those issued by higher-level institutions, resulting in lower PMC indices.

The mission of the institution also affects the policy’s focus and regulatory mechanisms. For example, the Ministry of Education’s policy (P6) emphasizes talent cultivation and the integration of industry and education, which aligns with its mission to promote educational development and support the transformation of research outcomes into practical applications (David et al., 2024) [23]. However, this focus may lead to a lack of emphasis on other important aspects such as data security and legal binding, which are critical for the effective implementation of AI policies. Similarly, policies issued by the State Internet Information Office (e.g., P13) prioritize data security and the innovative application of AI, reflecting its mission to regulate and promote the development of internet-related technologies. Yet, these policies may not cover other important areas such as talent cultivation and long-term sustainability, which are also essential for the comprehensive development of AI.

In conclusion, the PMC index status is indeed influenced by the mission of the institution. Higher-level institutions with broader missions tend to produce more comprehensive and strategically oriented policies, while specific ministries or departments may focus more narrowly on their respective domains. This observation suggests that the mission of the institution plays a significant role in shaping the characteristics and effectiveness of AI policies. Future policy-making should consider the mission of the issuing institution and strive to balance comprehensive guidance with specific regulatory needs to enhance the overall effectiveness of AI policies.

### 5.2. Multi-dimensional evaluation of policies

Despite the introduction of many AI-related policies, there are still insufficiencies in terms of legal protection and implementation. Many policies lack sufficient legally binding and effective enforcement mechanisms, leading to unsatisfactory implementation. For example, P1, which relies more on industry regulations and government initiatives, has weaker legal forces and faces greater implementation challenges compared to the more successful AI policy in Europe that incorporates AI ethical issues and data security into a rigorous legal framework through legislation (Niklas and Dencik, 2024) [4]. Most of the AI policies analyzed in this study are still only normative documents and industry regulations, and the lack of legal security and enforcement limits compliance and transparency in the development of AI technology.

In terms of ethical issues, current policies remain inadequate in addressing ethical risks. Many policies lack effective regulatory mechanisms for technology misuse and privacy violations. For instance, P8 proposes ethical norms but does not effectively promote the construction of relevant regulatory and technology assessment systems. Evangelista (2025) [9] mentions the ethical controversies triggered by the application of generative AI technology in the academic field, especially concerns about academic integrity, and suggests that policies should propose more specific regulatory measures. However, the ethical oversight and technology assessment mechanisms of many policies are still in their infancy and fail to adequately consider the socio-ethical risks and privacy concerns of AI applications.

The rapid development of AI technology requires close collaboration and support among various fields. Although many current policies encourage cross-sectoral collaboration, they mainly focus on short-term goals and lack long-term mechanisms. For example, P4 emphasizes cross-sectoral collaboration but is mainly concerned with short-term goals (Yang and Huang, 2022) [3]. Policies such as P1 and P2 cover issues such as data privacy and safety but have major deficiencies in specific cross-sectoral collaboration frameworks and implementation rules, which may result in asymmetric information and poor coordination between sectors, affecting the overall effectiveness of the policies.

While current AI policies focus on technological innovation and economic benefits, they pay little attention to the long-term social and environmental impacts of technology. Most policies only mention environment and environmental protection in the context of “AI+”, but they have not yet explained how to ensure resource sustainability while utilizing AI development, nor have they provided specific measures on how AI can play a role in environmental protection. For example, P10 is weak in predicting and responding to social inequality and environmental pollution that the technology may bring about (Danish and Senjyu, 2023) [15]. The current policy design focuses more on immediate technology application and economic growth, and neglects planning for long-term sustainability. Therefore, the lack of long-term sustainability in current policies has resulted in their failure to effectively anticipate and address the possible negative social impacts of technology.

## 6. Conclusions

In this study, we used text mining methods to analyze the word frequencies of 115 AI policies, extract the focus of China’s AI development, and construct a PMC policy index model, in combination with existing studies, to quantitatively evaluate the 14 AI policies released in China. The model is based on nine first-level indicators and several corresponding second-level indicators, covering policy type, timeliness, content, domain, evaluation, object, tool, focus, and effectiveness level dimensions, with the aim of assessing the degree of strength and weakness of China’s AI policies.

Based on the calculation of the PMC index, the 14 selected AI policies were categorized into three grades, including 1 grade I policy, 10 grade II policies, and 3 grade III policies. Among them, P4 is an excellent policy, with perfect performance in all seven indexes, and this policy can provide a reference for the formulation of subsequent policies. Overall, according to the calculation of the PMC index, China’s AI policies as a whole are on the good side and have played an important role in promoting technological innovation, economic growth, and social change, but there are still many challenges in terms of legal safeguards, ethical issues, cross-domain synergies, and sustainable development. This study provides a framework for the quantitative analysis of policy documents and summarizes the problems and directions for improvement of current Chinese AI policies, which can enable policymakers and researchers to obtain a more objective and comprehensive perception of the policies and provide a basis for their improvement.

## 7. Innovations, limitations and policy recommendations

### 7.1. Innovations

First, this study applied the Policy Model Consistency (PMC) methodology for the first time to the quantitative assessment of AI policies in China and constructed a comprehensive assessment index system. The system covers multiple dimensions, such as policy type, timeliness, content, domain, evaluation object, policy tool, focus, and effectiveness level. The introduction of this method effectively fills the gaps in the quantitative analysis of existing studies and significantly improves the scientific and objective nature of policy evaluations.

Second, in the selection of policy samples, this study adopts a systematic screening method to ensure the representativeness of the samples. In addition, high-frequency words were analyzed in the policy literature through text mining technology, revealing the core themes and focuses of the policies. This innovative approach not only enhances the in-depth understanding of policy content but also lays a solid foundation for subsequent quantitative analysis.

Third, this study combines a cross-country comparative perspective to deeply analyze the similarities and differences between China’s AI policies and those of developed countries in Europe and the United States, further revealing the development trends of global AI policies. This comparative study not only enriches the understanding of policy differences between countries but also provides important theoretical support for the optimization of China’s AI policy. Through the calculation and evaluation of the PMC index, this study empirically analyzes the advantages and disadvantages of different policies and puts forward targeted improvement suggestions, providing a scientific basis for policymakers.

Finally, this study provides an in-depth analysis of the shortcomings of China’s AI policies in terms of legal protection, ethical response, cross-domain synergy, and long-term sustainability, and proposes specific directions for improvement. The findings provide an important reference for future policy research and practice and promote the responsible development of AI technology.

### 7.2. Innovations

Although the policy samples selected in this study are representative, the sample size is relatively small and focuses mainly on policy documents at the national level, failing to adequately cover local policies and specific implementation programs at the industry level. Limitations of the sample may affect the breadth and accuracy of the assessment results. Therefore, future research should expand the scope of the sample to include more policy documents at different levels, fields, and regions, especially the analysis of local and industry-specific policies, which will help to enhance the representativeness of the study and the universality of the results and provide a more comprehensive perspective for policy assessment.

Although the Policy Model Consistency (PMC) approach provides a systematic framework for policy evaluation, it relies on binary assignment and high-frequency word analysis in its practical application, which may lead to oversimplification of policy content and loss of potential information. In particular, the complex and multidimensional characteristics of policies may not be fully presented by simplified quantitative means. Therefore, future research should explore the combination of qualitative analysis methods with PMC modeling, especially when dealing with the micro-mechanisms of the policy implementation process, which can capture the details and complexity of policy implementation more comprehensively and enhance the applicability and accuracy of PMC modeling.

### 7.3. Policy recommendations

Further strengthening of legal frameworks and ethical regulations. Governments should formulate AI-related laws and regulations to address potential risks and challenges in AI applications, especially in the context of globalization, where legal cooperation and regulatory coordination across borders are crucial. Referring to the policy approach of transnational co-creation proposed by Mathiyazhagan and La Fors (2023) [17], among others, it is important to strengthen legal binding on issues such as data privacy, algorithmic discrimination, and technology misuse. Simultaneously, the policy should clearly define the ethical standards and regulatory procedures for all types of AI applications to ensure that the technology applications are always in line with the social ethical requirements and that they can obtain legal social licenses. Additionally, a specialized regulatory body should be established to ensure that the technology is always under control during its use.

Cross-field cooperation and innovation should be strengthened to promote the realization of “AI +”. It should promote in-depth cooperation among academia, social organizations, and enterprises; encourage the joint establishment of research centers; jointly carry out research and development projects on AI technology; and deepen the integration of industry, academia, and research. In particular, the sharing and integration of information and resources from multiple parties should be strengthened in various areas, including technological, social, and ethical supervision. In particular, the government should encourage the establishment of cross-sectoral working groups and industry alliances to promote the transformation of AI industry achievements and ensure that policies are effectively supported and implemented in multiple ways. Additionally, public participation and social co-governance should be promoted. Through multi-channel information dissemination and publicity campaigns, strengthening society’s understanding and knowledge of AI technology, increasing public acceptance and participation in the policy, and enhancing the public’s information capability and AI literacy. In the future, AI policies should focus on internationalization and global cooperation, especially in the context of increasingly fierce global competition in AI technology, which should strengthen cooperation and communication with other countries, promote the construction and cooperation of the global AI ecosystem, and enhance China’s influence in the global science and technology field.

Emphasize sustainable development and long-term goals. To realize the sustainable development of the AI industry and the long-term interests of society, policymakers should encourage technology enterprises to pay attention to environmental protection and social responsibility during the innovation process and incentivize them to research and develop AI technologies in line with the goal of sustainable development through tax incentives and financial subsidies.

Strengthening ethics education and social responsibility training. Policies should encourage and promote the construction of relevant educational systems to ensure ethical application of AI technology. In particular, the cultivation of ethical awareness and social responsibility must be strengthened among AI developers and applicators. Through the formulation of corresponding education policies, ethics education should be incorporated into the professional curriculum of AI to cultivate AI talent with ethical thinking and a sense of social responsibility. In addition, the government can promote ethical training in enterprises so that technology developers and users will adhere to ethical principles in the application of technology and reduce the possibility of technological abuse.

## Supporting information

S1 File Matrix. (XLSX)
